# From geriatric assessment to inflammation. A pilot, observational, study about frailty components in older patients with persistent atrial fibrillation

**DOI:** 10.1016/j.ijcha.2024.101558

**Published:** 2024-11-21

**Authors:** Stefano Fumagalli, Giulia Ricciardi, Claudia Di Serio, Elisa Berni, Giancarlo La Marca, Giuseppe Pieraccini, Riccardo Romoli, Emanuele Santamaria, Giulia Spanalatte, Camilla Cagnoni, Arianna Tariello, Giada Alla Viligiardi, Agostino Virdis, Igor Diemberger, Andrea Ungar, Niccolò Marchionni

**Affiliations:** a Department of Experimental and Clinical Medicine, Geriatric Intensive Care Unit, University of Florence and AOU Careggi, Florence, Italy; b Mass Spectrometry Centre (CISM), University of Florence, Florence, Italy; c Newborn Screening, Clinical Chemistry and Pharmacology Lab, Meyer Children’s Hospital IRCCS, Florence, Italy; d Geriatrics Unit, Department of Clinical and Experimental Medicine, University of Pisa, Pisa, Italy; e Department of Medical and Surgical Sciences, DIMEC, University of Bologna, Bologna, Italy; f Cardiology Unit, IRCCS Policlinico di S. Orsola, Bologna, Italy

**Keywords:** Atrial fibrillation, Frailty, Older patients, Inflammation, Physical performance, Acylcarnitines

## Abstract

**Background:**

Atrial fibrillation (AF) is the most common arrhythmia diagnosed at an older age. AF is associated with frailty, a condition possibly justifying the higher rate of complications and mortality in aged individuals. This study was aimed at describing the characteristics correlated to frailty in older AF subjects.

**Methods:**

After having excluded a < 3 months major surgery procedure, cancer or other conditions associated with activation of inflammation, and a life expectancy < 12 months, we consecutively enrolled patients ≥ 65 years with persistent AF. They underwent a Comprehensive Geriatric Assessment evaluation. In particular, Mini-Mental State Examination, 15-item Geriatric Depression Scale and Short-Physical Performance Battery (SPPB) described, respectively, cognitive profile, depressive symptoms and physical performance. A venous blood sample was collected to measure interleukin-6 (IL-6; marker of low-grade inflammation) and acylcarnitines, expression of mitochondrial dysfunction and abnormal energy production.

**Results:**

Overall, 49 patients (mean age: 76 ± 6 years; women 30.6 %) were studied. Cluster analysis described two different patterns; the second (N = 18, 36.7 %), when compared to the first one (N = 31, 63.3 %), was characterized by a worse phenotype, identified by the simultaneous presence of lower body mass index, higher CHA_2_DS_2_-VASc score (index of clinical complexity), worse SPPB functional performance, and high IL-6 levels. Second cluster patients had a higher concentration of 13 of the 35 acylcarnitines evaluated and increased 5-year mortality. All these features can outline a frail condition.

**Conclusions:**

Body size, clinical complexity, physical performance and low-grade inflammation seem to rapidly and adequately describe frailty.

## 1 Introduction

Atrial fibrillation (AF) is the most common sustained arrhythmia diagnosed at an advanced age. Recently, a growing interest originated concerning the link between AF and frailty, a multi-factorial syndrome caused by the reduction of physiological reserve and of capability to resist stressful events, characterized by changes in energy production, distribution, and utilization [1], [2]. The presence of comorbidities and disability variably contribute to the frail phenotype [3]. In older subjects, the pathophysiology of AF is complex. Chronic low-grade inflammation, frequently found during ageing, and known as “inflamm-ageing”, could be a condition common to frailty and AF itself [4]. Accordingly, this pilot study was aimed at evaluating if some clinical characteristics and interleukin-6 (IL-6), a marker of inflammation, could be useful to describe frailty in older patients with the arrhythmia. Indeed, IL-6 was recently shown to be associated with Frailty Index in home-dwelling individuals aged > 70 years [5], and it resulted independently correlated with cardiovascular events and mortality in older adults [6]. To further evaluate the plausibility of our findings, in the clusters of patients which were outlined, we studied the rate of all-cause mortality and mitochondrial dysfunction, identified by higher levels of acylcarnitines, which had revealed to be important diagnostic biomarkers [7]. Definitely, it is important to better characterize frailty pathophysiology in subjects with AF because the condition determines oral anticoagulants undertreatment and a lower recourse to a rhythm control strategy, significantly influencing the AF-CARE pathway (Comorbidity and risk factor management; Avoid stroke and thromboembolism; Reduce symptoms by rate and rhythm control; Evaluation and dynamic reassessment) proposed by current guidelines [8]. Last, we aimed at assessing feasibility and acceptability of the protocol in order to improve study design and eventually to enroll a higher number of patients.

## 2 Methods

### 2.1 Patients’ evaluation and treatment

Consecutive older outpatients (age ≥ 65 years) with persistent AF directed to a rhythm-control strategy program of the arrhythmia were prospectively enrolled in the study, provided their consent to participate to the protocol [9], which was approved by local ethical committee and was conform to the Declaration of Helsinki. Each subject was evaluated after the admission in a Day-Hospital setting to be submitted to electrical cardioversion (ECV) of the arrhythmia. There were no exclusion criteria other than a < 3 months major surgery procedure, the presence of cancer or other diseases associated with an overt activation of inflammation, and a life expectancy < 12 months. All patients underwent venous blood sampling in a fasting condition. After a cardiologic visit, all subjects were evaluated also using the Comprehensive Geriatric Assessment tools, in particular, the Mini-Mental State Examination (MMSE; cognitive function; score range from 0 to 30, best performance) [10], the 15-item version of the Geriatric Depression Scale (GDS; depressive symptoms; score range from 0 to 15, major burden) [11] and the Short-Physical Performance Battery (SPPB; physical function; score range from 0 to 12, best physical performance) [12]. Then, ECV was performed using a synchronized biphasic shock (mean energy value: 175 Joule) after the administration of propofol (1 mg/kg of body weight) to obtain a 5–10 min length anaesthesia. Patients were then discharged home after 2 h from the procedure [9].

### 2.2 Laboratory measures

Acylcarnitines, derived from fatty acid metabolism and involved in the cellular pathways producing energy, are differentiated in short (C2-5), medium (C6-12), long (C13-20) and very-long chain (>C20) molecules, based on the number of the carbon atoms in the acyl-chains [7]. A plasma sample was collected and stored at −80 °C until acylcarnitines and IL-6 levels determination, using, respectively, mass spectrometry techniques and ELISA kits (R&D Systems™ Human IL-6 Quantikine HS ELISA, Bio-Techne, MN, USA).

### 2.3 Statistical analysis

IBM SPSS (version 29) was used to describe the study population and to perform all statistical analysis procedures. Categorical variables are expressed as raw numbers and percentages, continuous variables as mean and standard deviation. To study frailty components, we decided to use cluster analysis that allows, starting from a set of variables, to separate patients into different groups (i.e., clusters) to explore distinctive behaviours, possibly representing a basis for further, more detailed, analyses. Ideally, a cluster is formed by a set of components whose reciprocal distances are lower than the distances separating them from components of other clusters. Accordingly, a cluster is a region with a high density of elements separated from others by low density areas [13]. In conclusion, cluster analysis may be considered an exploratory tool aiming at delineating natural groupings otherwise not apparent. We forced into the models the CHA_2_DS_2_-VASc score, as an indicator of clinical complexity [9], the SPPB score, correlated to physical performance, and IL-6, as a marker of low-grade inflammation, iteratively testing the effects of sex, body mass index (BMI), heart rate (HR), systolic arterial pressure (SAP), MMSE and GDS, for cognitive function and depressive symptoms, and of living alone. Cluster analysis was performed with a log-likelihood measure of distance applying a Schwarz’s Bayesian criterion. The model with the highest performance for cohesion and separation was selected. Differences between clinical characteristics of the two resulting clusters were assessed for continuous variables with the Student’s *t*-test or the Mann-Whitney test (in the case of a not normal distribution), and with the chi-square test for discrete variables. Kaplan-Meier analysis was used to assess differences in all-cause mortality during the follow-up. The associated hazard ratio (HR), with the related 95 % confidence intervals (95 %CI), was obtained with a univariate Cox regression analysis model. As previously reported, cluster analysis was performed using the CHA_2_DS_2_-VASc score as an indicator of clinical complexity [9]. Because of the recent publication of the 2024 ESC guidelines for the management of AF [8], we ran a second model substituting the CHA_2_DS_2_-VASc with the CHA_2_DS_2_-VA score, now recommended as the preferred tool to assess individual thromboembolic risk, to check for the consistency of our conclusions. Because of the novelty of this approach to frailty, sample size estimation was not performed. However, an indicatively only retrospective power analysis was run on the most important variables evidenced by cluster definition. In all cases, a two-sided p-value < 0.05 indicated statistical significance.

## 3 Results

### 3.1 Patients’ description

Fifty consecutive patients were enrolled; no one refused to participate. However, due to technical reasons, one subject was excluded from the analysis. Mean age was 76 ± 6 years (women – N = 15, 30.6 %). Clinical characteristics of patients are reported in Table 1. Hypertension was the most frequent comorbid condition. The prevalence of coronary artery disease and chronic heart failure were, respectively, 26.5 and 34.7 %; study population showed normal mean values of left ventricular end-diastolic diameter and ejection fraction, and a dilated left atrium. The proportion of patients with diabetes and chronic renal failure was 14.3 and 20.4 %. Overall, cognitive function was preserved and depressive symptoms were low. Amiodarone was the most frequently used anti-arrhythmic agent. Heart rate, systolic and diastolic arterial pressure were well controlled.

**Table 1:** Clinical characteristics of patients.

	**All patients** **(N = 49)**	**Cluster 1** **(N = 31)**	**Cluster 2** **(N = 18)**	**P**
**Age (years)**	76 ± 6	74 ± 6	79 ± 6	0.018
**Women (N, %)**	15 (30.6)	9 (29.0)	6 (33.3)	0.141
**Height (cm)**	171 ± 9	172 ± 9	169 ± 8	0.256
**Weight (Kg)**	77 ± 12	80 ± 11	73 ± 14	0.021
**Living alone (N, %)**	10 (20.4)	4 (12.9)	6 (33.3)	0.141
**MMSE (score)**	28.1 ± 2.2	28.8 ± 1.4	26.9 ± 2.8	0.011
**GDS (score)**	2.8 ± 2.3	2.1 ± 1.5	3.7 ± 2.8	0.047
**Current smoker (N, %)**	4 (8.2)	4 (12.9)	0 (0.0)	0.282
**Hypertension (N, %)**	42 (85.7)	26 (83.9)	16 (88.9)	0.701
**Dyslipidemia (N, %)**	21 (42.9)	12 (38.7)	9 (50.0)	0.553
**Diabetes (N, %)**	7 (14.3)	4 (12.9)	3 (16.7)	0.697
**Hyperuricemia (N, %)**	15 (30.6)	5 (16.1)	10 (55.6)	0.009
**CAD (N, %)**	13 (26.5)	7 (22.6)	6 (33.3)	0.508
**CHF (N, %)**	17 (34.7)	10 (32.3)	7 (38.9)	0.758
**PAD (N, %)**	10 (20.4)	7 (22.6)	3 (16.7)	0.726
**Stroke/TIA (N, %)**	5 (10.2)	2 (6.5)	3 (16.7)	0.342
**COPD (N, %)**	9 (18.4)	5 (16.1)	4 (22.2)	0.708
**CKD (N, %)**	10 (20.4)	4 (12.9)	6 (33.3)	0.141
**RAS blockers (N, %)**	41 (83.7)	26 (83.9)	15 (83.3)	1.000
**β-blockers (N, %)**	38 (77.6)	26 (83.9)	12 (66.7)	0.286
**Diuretics (N, %)**	31 (63.3)	18 (58.1)	13 (72.2)	0.372
**Statins (N, %)**	23 (46.9)	12 (38.7)	11 (61.1)	0.151
**Amiodarone (N, %)**	23 (46.9)	11 (35.5)	12 (66.7)	0.043
**Digoxin (N, %)**	17 (34.7)	9 (29.0)	8 (44.4)	0.355
**HR (bpm)**	76 ± 15	74 ± 13	80 ± 18	0.217
**SAP (mmHg)**	132 ± 20	134 ± 21	130 ± 20	0.579
**DAP (mmHg)**	80 ± 11	80 ± 10	80 ± 14	0.891
**LAD (mm)**	55 ± 6	53 ± 6	58 ± 6	0.031
**LVEDD (mm)**	52 ± 8	52 ± 7	51 ± 10	0.767
**LVESD (mm)**	34 ± 10	34 ± 8	35 ± 13	0.787
**LVEF (%)**	59 ± 12	60 ± 10	56 ± 14	0.281
**Hemoglobin (g/dL)**	13.5 ± 1.6	13.6 ± 1.5	13.5 ± 1.6	0.968
**WBC (N.10^-3^/mm^3^)**	6.6 ± 2.1	6.3 ± 1.9	7.0 ± 2.3	0.307

CAD: coronary artery disease; CHF: chronic heart failure; CKD: chronic kidney disease; COPD: chronic obstructive pulmonary disease; GDS: 15-item Geriatric Depression Scale; HR: heart rate; LAD: left atrium diameter; LVEDD/LVESD: left ventricle end-diastolic/systolic diameter; LVEF: left ventricular ejection fraction; MMSE: Mini-Mental State Examination; PAD: peripheral artery disease; RAS: renin-angiotensin system; SAP/DAP systolic/diastolic arterial pressure; WBC: white blood cells.

**Fig. 1:**
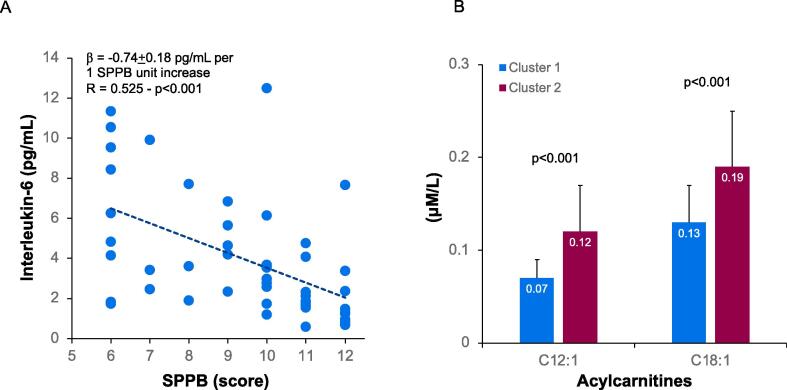
(Panel A) Graphical representation of the inverse association between IL and 6 and SPPB scores; the better the physical performance, the lower the cytokine concentration. (Panel B) Different levels of C12:1 and C18:1 acylcarnitines by cluster of older AF patients. The concentration is significantly higher in subjects with the worse phenotype.

IL-6 levels were inversely associated with SPPB score (Fig. 1, panel A), but not with BMI (p = 0.189) and the CHA_2_DS_2_-VASc score (p = 0.070).

### 3.2 Cluster analysis of patients

After having iteratively tested the effects of sex, BMI, HR, SAP, MMSE and GDS, and living alone, together with those of CHA_2_DS_2_-VASc and SPPB scores, and of IL-6, the best fitting cluster analysis model, with a good performance in terms of cohesion and separation, allowed to identify two different phenotypic profiles in our population, the first composed by 31 subjects (63.3 %) and the second by 18 (36.7 %). The centroid values of the first cluster were consistent with a better condition than those of the second one for each of the four determinants of the model, in particular, for BMI (27.1 ± 4.3 vs. 25.5 ± 4.0 Kg/m^2^), CHA_2_DS_2_-VASc score (3.4 ± 1.5 vs. 4.3 ± 1.6), SPPB (10.9 ± 1.0 vs. 7.1 ± 1.3) and IL-6 concentration (2.6 ± 1.7 vs. 6.2 ± 3.5 pg/mL). SPPB was the most relevant variable for classification (predictor importance, PI: 1.0), followed by IL-6 concentration (PI: 0.3), CHA_2_DS_2_-VASc score (PI: 0.1) and BMI (PI: 0.1). Overall, only indicatively, taking into consideration present cluster composition (#1 − N = 31, #2 − N = 18), a retrospective power analysis assuming the differences of the mean of SPPB score and of IL-6 gave values, respectively, > 0.98 (for a p value < 0.001) and > 0.86 (for a p value < 0.01).

When compared to cluster 1 patients, those in cluster 2 were older, had a lower body weight, a reduced cognitive performance and a higher burden of depressive symptoms. The prevalence of hyperuricemia was more frequently observed. Also, amiodarone was more often prescribed and left atrium diameter was larger in cluster 2 patients (Table 1).

If substituting the CHA_2_DS_2_-VASc with the CHA_2_DS_2_-VA score in clusters characterization, differences between the two phenotypes were still present in CHA_2_DS_2_-VA (2.5 ± 1.1 vs. 4.1 ± 1.5), SPPB (11.3 ± 0.7 vs. 8.1 ± 1.9) and IL-6 concentration (2.0 ± 1.2 vs. 5.3 ± 3.3 pg/mL).

### 3.3 Acylcarnitines concentration by patients’ cluster

For 13 of the 35 (37.1 %) analysed acylcarnitines, a higher concentration was found in cluster 2 patients, those with the worse phenotype. Differences were observed among short, medium and long chain molecules, with the greatest regarding C12:1 (p < 0.001) and C18:1 (p < 0.001) acylcarnitines (Fig. 1, panel B). The concentration by cluster was not different for the other 22 molecules.

### 3.4 All-cause mortality by patients’ cluster

**Fig. 2:**
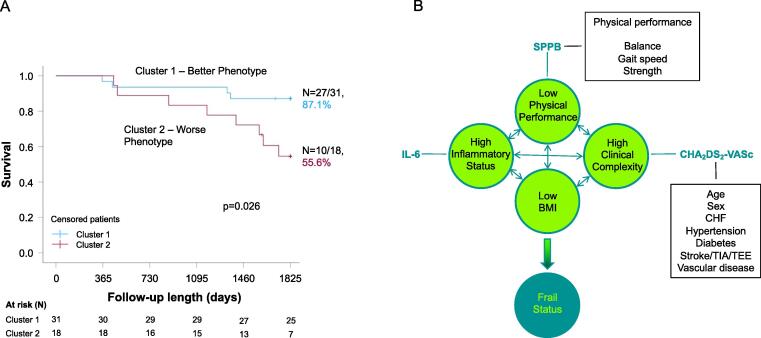
(Panel A) Kaplan-Meier analysis of survival by cluster; at the follow-up evaluation, patients with the better phenotype – blue line − are characterized by lower all-cause mortality than the worse phenotype subjects – burgundy line. (Panel B) The four variables associated with cluster definition (i.e., low BMI, CHA_2_DS_2_-VASc and SPPB scores, and IL-6), also reciprocally interacting, could cause the frail status in older patients with persistent atrial fibrillation. Abbreviations. BMI: body mass index; CHF: chronic heart failure; IL-6: interleukin-6; TIA: transient ischaemic attack; SPPB: Short-Physical Performance Battery; TEE: thrombo-embolic event.

At the 5-year follow-up, 8 out of 18 patients (44.4 %) had died in cluster 2, a proportion significantly higher than that observed in cluster 1 (N = 4/31, 12.9 %), with mean survival times of 1530 ± 108 and 1702 ± 65 days, respectively (p = 0.026; Fig. 2, panel A). At the Cox regression analysis, when compared to cluster 1, the HR for mortality for the cluster 2 subjects was 3.90 (95 %CI: 1.17–12.99).

## 4 Discussion

The results of the present pilot study helped to identify two clusters of older AF patients. The lower BMI, attributable to reduced body weight, as in frailty definition [3], [14], the greater clinical complexity (CHA_2_DS_2_-VASc score), the worse physical performance (SPPB score), and the higher concentration of IL-6 allowed to delineate the worse phenotypic profile of our study population. Interestingly, a recent *meta*-analysis of 57 studies showed that the mean IL-6 level in healthy subjects is 5.2 pg/mL (95 %CI: 4.6–5.7), with an increase of 0.05 pg/mL for each year of age [15]. Hence, the concentration we observed in our cluster with the worse phenotype was only slightly higher (mean level: 6.2 pg/mL) than that observed in the absence of disease. At this regard, it is to be considered that inflamm-ageing is a chronic, systemic, low-grade, pro-inflammatory status, in which long-time persisting subclinical inflammatory stimuli are the biologic background promoting the development of age-related frailty conditions [16]. To the worse phenotypic description that we delineated corresponded a higher concentration of many of the acylcarnitines we studied, related to a negative effect on mitochondrial physiology, energy metabolism and contractility, due to the interaction with cell membranes and Ca^2+^ handling [17]. Regarding those with the higher differences between the two clusters of patients we identified, evidence suggest that C12:1 is associated with mitochondrial dysfunction in diabetic subjects, and C18:1 is correlated with all-cause mortality and hospitalizations in the presence of heart failure, and with ischemia–reperfusion injuries [7]. In particular, concerning AF, we found that the levels of C18:1 acylcarnitine, in vitro associated with an arrhythmogenic action [17], were particularly elevated in cluster 2 patients. Furthermore, we had previously found that acylcarnitines and lower physical performance were correlated with IL-6 concentration [18]. To be able to measure these molecules at the bedside could allow to improve patients’ stratification and to more carefully monitor the effects of interventions in such a complex population. Moreover, our cluster 2 subjects had higher uric acid levels, a finding that had been already associated with an increased concentration of low-grade inflammation markers [19]. Last, patients with the worse phenotype had a higher mortality during the follow-up. The related HR (3.90; 95 %CI: 1.17–12.99) was slightly greater than that observed in a *meta*-analysis of 10 studies evaluating the impact of frailty on AF patients’ prognosis (RR = 2.77; 95 %CI: 1.68–4.57) [20]. In this sense, cluster 2 characterization could promote some interventions aimed at improving subjects’ at-risk profile. In particular, a recent report of the American Heart Association evidenced that an inadequate physical activity and a poor diet are present, respectively, in > 60 and > 45 % of subjects older ≥ 80 years [21]. Also, a rhythm-control strategy of the arrhythmia has been recently associated not only with the reduction of unfavourable cardiovascular outcomes [22], but also with improved brain perfusion [23] and a lower incidence of dementia [24]. These findings sustain the hypothesis that an integrated intervention supported by the geriatric and the AF-CARE [8] approaches could exert a positive effect on survival.

Some of the current knowledge gaps about frailty are centred on the lack of a research-oriented, shared, operative, definition of the condition. Accordingly, the ideal instrument to evaluate frailty should be simple, quantitative, objective, multidimensional and capable of providing a consistent, valid, and reproducible description of this particular condition [25]. Indeed, our patients characterized by the worse phenotype, associated with abnormalities in energy handling and a lower survival during the follow-up, seem to adequately fit to frailty representation (Fig. 2, panel B) [25].

### 4.1 Limitations of the study

Even if using the Comprehensive Geriatric Assessment tools and advanced laboratory techniques, we studied a small number of cases in a single centre. We cannot exclude that not homogeneous local protocols in diagnostic and therapeutic strategies for older AF patients’ management, and different analytic techniques for the measurement of molecules could have led to diverse results in other centres. Furthermore, it was impossible to run a multivariable Cox analysis model to evaluate the independent association of clusters with outcomes adjusting for the covariates we included in the database. Accordingly, differences in some clinical, instrumental and therapy variables remain only descriptive. However, our findings are clinically plausible and should be considered as hypothesis generating. Indeed, the coexistence in the cluster analysis model of BMI, with the CHA_2_DS_2_-VASc and SPPB scores, allowed to simultaneously verify the effects of body size, of some of the most important comorbidities and of physical function. To underline the consistency of our results, also the CHA_2_DS_2_-VA score, when substituting the CHA_2_DS_2_-VASc score [8], maintained its association with clusters definition. We enrolled only Caucasian patients; our conclusions could not be extended to other ethnicities. Last, our findings could have been different in the case of patients with paroxysmal or permanent forms of the arrhythmia, or in the case of subjects with persistent AF not directed to ECV.

### 4.2 Conclusions

The findings of this pilot study seem to suggest that, in older patients with persistent AF, frailty is associated with body size, clinical complexity, physical performance and low-grade inflammation. If these results will be confirmed by further observations with a larger sample size, a strategy oriented to control or to revert the condition could be designed. At this regard, targeted protocols, aimed at preserving or increasing muscular mass, improving metabolic conditions, reducing the exposition to risk factors and optimizing the medical management of AF could reveal useful to improve the prognosis of this segment of population, the most exposed to the severe and disabling complications of the arrhythmia.

## Data availability statement

Because the original project is still actively enrolling patients, the data underlying this article will be shared only on reasonable request to the corresponding author.

## CRediT authorship contribution statement

**Stefano Fumagalli:** Writing – review & editing, Writing – original draft, Methodology, Investigation, Funding acquisition, Formal analysis, Data curation, Conceptualization. **Giulia Ricciardi:** Writing – review & editing, Investigation, Data curation. **Claudia Di Serio:** Writing – review & editing, Project administration, Methodology, Data curation. **Elisa Berni:** Writing – review & editing, Project administration, Data curation. **Giancarlo La Marca:** Writing – review & editing, Resources, Methodology, Investigation, Data curation. **Giuseppe Pieraccini:** Writing – review & editing, Validation, Resources, Methodology, Investigation. **Riccardo Romoli:** Writing – review & editing, Software, Methodology, Formal analysis. **Emanuele Santamaria:** Writing – review & editing, Investigation, Data curation. **Giulia Spanalatte:** Writing – review & editing, Investigation, Data curation. **Camilla Cagnoni:** Writing – review & editing, Investigation, Data curation. **Arianna Tariello:** Writing – review & editing, Investigation, Data curation. **Giada Alla Viligiardi:** Writing – review & editing, Validation, Investigation. **Agostino Virdis:** Writing – review & editing, Resources, Methodology, Investigation, Funding acquisition. **Igor Diemberger:** Writing – review & editing, Resources, Methodology, Investigation, Funding acquisition. **Andrea Ungar:** Writing – review & editing, Resources, Funding acquisition. **Niccolò Marchionni:** Writing – review & editing, Resources, Methodology, Funding acquisition.

## Funding

This work was supported by the Italian Ministry of University and Research and Next Generation EU (Project of Relevant National Interest, PRIN - 2022L9NPKH).

## Declaration of competing interest

The authors declare that they have no known competing financial interests or personal relationships that could have appeared to influence the work reported in this paper.
